# Endoscopic Ultrasound-Guided Drainage of Pancreatic Fluid Collections: Not All Queries Are Already Solved

**DOI:** 10.3390/medicina60020333

**Published:** 2024-02-16

**Authors:** Cecilia Binda, Stefano Fabbri, Barbara Perini, Martina Boschetti, Chiara Coluccio, Paolo Giuffrida, Giulia Gibiino, Chiara Petraroli, Carlo Fabbri

**Affiliations:** 1 Gastroenterology and Digestive Endoscopy Unit, AUSL Romagna, Forlì-Cesena Hospitals, 47121 Forlì, Italy; 2 Gastroenterology and Digestive Endoscopy Unit, Azienda ULSS 5, 45100 Rovigo, Italy; 3 Department of Medical and Surgical Sciences, University of Bologna, 40126 Bologna, Italy

**Keywords:** walled-off pancreatic necrosis, pancreatic fluid collections, EUS-guided drainage, step-up approach, LAMS, double pigtail plastic stents, SEMS, direct endoscopic necrosectomy

## Abstract

Pancreatic fluid collections (PFCs) are well-known complications of acute pancreatitis. The overinfection of these collections leads to a worsening of the prognosis with an increase in the morbidity and mortality rate. The primary strategy for managing infected pancreatic necrosis (IPN) or symptomatic PFCs is a minimally invasive step-up approach, with endosonography-guided (EUS-guided) transmural drainage and debridement as the preferred and less invasive method. Different stents are available to drain PFCs: self-expandable metal stents (SEMSs), double pigtail stents (DPPSs), or lumen-apposing metal stents (LAMSs). In particular, LAMSs are useful when direct endoscopic necrosectomy is needed, as they allow easy access to the necrotic cavity; however, the rate of adverse events is not negligible, and to date, the superiority over DPPSs is still debated. Moreover, the timing for necrosectomy, the drainage technique, and the concurrent medical management are still debated. In this review, we focus attention on indications, timing, techniques, complications, and particularly on aspects that remain under debate concerning the EUS-guided drainage of PFCs.

## 1. Introduction

Acute pancreatitis is the most common gastrointestinal disease that requires urgent hospitalization. Most cases (80%) follow a mild, self-limiting course [1]. However, severe necrotizing pancreatitis, despite improved management, continues to carry a poor prognosis and is linked to high rates of early organ failure (38%), interventional requirement (38%), and death (15%) [2]. Pancreatic fluid collections (PFCs) are well-known complications, including walled-off pancreatic necrosis (WOPN), posing significant management challenges. Infection of these collections drastically worsens prognosis, elevating morbidity and mortality rates [3]. A “step-up” minimally invasive approach has emerged as the preferred method for managing infected pancreatic necrosis (IPN), replacing open surgical necrosectomy [4]. This approach, involving endosonography-guided transmural drainage and potential mechanical debridement, offers reduced multi-organ failure, external pancreatic fistulae, and other complications, along with shorter hospital stays and improved quality of life. According to the revised Atlanta classification (2012) [5], the severity of acute pancreatitis can be categorized as mild, moderately severe, or severe based on the absence of complications, presence of complications with transient (<48 h), or persistent (>48 h) organ failure, respectively. Severe cases carry a mortality rate of 20–40% and demand a multidisciplinary approach due to high complication and mortality rates [6].

The most common local complications of acute pancreatitis involve the formation of pancreatic or peripancreatic fluid collections (PFCs) resulting from pancreatic injury due to the release of proteolytic fluid from the pancreas into the adjacent cavity of the peritoneum. According to the revised Atlanta classification (2012) [5], PFCs can be categorized into four types of PFCs, based on the timing of appearance (< or >4 weeks) and the presence of solid necrosis: acute peripancreatic collections, pancreatic pseudocysts, acute necrotic collections (ANCs), and walled-off pancreatic necrosis (WOPN).

In patients with infected necrosis or symptomatic pancreatic fluid collections (PFCs), the European Society of Gastrointestinal Endoscopy (ESGE) guidelines [7] and the American Gastroenterological Association (AGA) guidelines [8] recommend drainage and/or debridement of pancreatic necrosis. Historically, open necrosectomy involving surgical debridement and postoperative lavage was considered the gold standard treatment for infected necrosis. However, over the past 15 years, there has been a gradual shift from traditional open surgery toward minimally invasive interventions, adopting the ‘step-up approach’. This multistep approach involves a progressive transition from less invasive to more invasive procedures if needed. Currently, EUS-guided drainage stands out as the gold standard first-line treatment for symptomatic or infected WOPN. In this evolving landscape, this up-to-date review primarily focuses on indications, timing, techniques, and aspects that remain under debate concerning EUS-guided drainage and the endoscopic management of symptomatic pancreatic fluid collections.

## 2. Indications for Drainage of PFCs

**Figure 1:**
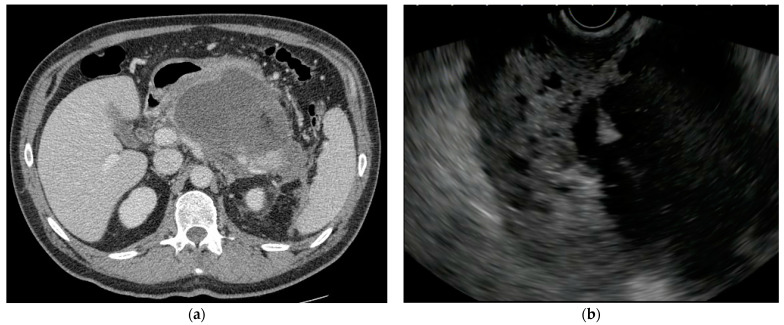
Contrast-enhanced Computed Tomography (CE-CT) (**a**) and EUS (**b**) appearance of a large WOPN located in the body-tail of the pancreas.

The approach to symptomatic or infected pancreatic necrosis and the correct timing of intervention depends on the clinical conditions of the patient and the maturation of the necrotic collection. Therefore, imaging is mandatory to determine the optimal management strategy. The magnetic resonance (MRI) and contrast-enhanced computed tomography (CE-CT) appearances of WOPN are those of irregular fluid collections that form in the region of pancreatic necrosis, often extending into the peripancreatic space [9]. CE-CT best detects parenchymal pancreatic necrosis 72 h after symptom onset; before that time, it may underestimate or miss the presence of necrosis [10]. CE-CT is the first-line imaging modality used to assess the morphological features of ANP (Figure 1a), while MRI is preferred when iodinated contrast medium is contraindicated (e.g., poor renal function or allergy to iodinated contrast) or when radiation exposure has to be avoided (e.g., pregnant women). MRI and magnetic resonance cholangiopancreatography (MRCP) offer superior interobserver agreement and better discrimination of solid debris from fluid [11]. Endoscopic ultrasound (EUS), although more invasive, accurately evaluates WOPN content (Figure 1b).

According to ESGE guidelines [8], indications for invasive (radiological, endoscopic, or surgical) interventions in ANP are as follows:

Proven or suspected infected pancreatic necrosis (IPN). IPN is diagnosed when a Gram stain or culture of (peri)pancreatic tissue from percutaneous, endoscopic, or surgical drainage reveals the presence of bacteria and/or fungi. The presence of extraluminal gas in the pancreas and/or peripancreatic tissues on CT scans, as well as clinical signs of sepsis (such as a temperature of greater than 38 °C, symptoms of persistent systemic inflammatory response syndrome (SIRS), and worsening or no change in clinical state), might lead to the suspicion of IPN. In a Dutch post hoc retrospective analysis of a prospective multicenter database (208 patients) by van Baal et al. [12], the added value of routine fine-needle aspiration (FNA) in addition to clinical and imaging signs of infection in patients who underwent intervention for suspected INP was evaluated. Infection was confirmed in 80% of 92 patients with clinical deterioration (clinical group), in 94% of 88 patients with gas bubbles on CT scan (imaging group), and in 86% of 28 patients of the FNA group (*p* = 0.07). Therefore, ESGE guidelines [7] recommend against routine percutaneous FNA of (peri)pancreatic collections if there are clear clinical and radiological signs of IPN because of its limited therapeutic application [13], the high rate of false-negative and false-positive rates (25% and 15%, respectively) [12], and the risk of procedure-related complications (e.g., bleeding, perforation, iatrogenic infection) [14]. Another biological marker of IPN is procalcitonin. According to a recent prospective study by Samanta et al. [15], baseline PCT > 1.0 ng/mL is associated with poor outcome, with higher need for ICU (*p* = 0.001) and mortality (*p* = 0.044); PCT > 2.25 ng/mL and failure in reduction in PCT levels to <60% of its baseline at day 7 post-intervention can identify high-mortality-risk patients. The microbiological spectrum of 55 IPNs undergoing endoscopic draining and necrosectomy was assessed in a retrospective analysis [16]. A total of 27 patients (49%) at the index endoscopy had a single microbial species detected, whereas 28 patients (51%) had polymicrobial results. The most common microbiological findings were found in the form of Enterococci (45%), which included *E. faecium* and *E. faecalis*; Enterobacteriaceae (42%), which included strains of *Escherichia coli* and *Klebsiella pneumoniae* that produced extended-spectrum beta-lactamase (ESBL); and fungi (22%), which included *Candida albicans* and *Candida glabrata*. These were followed by non-hemolytic *Streptococci*, coagulase-negative *Staphylococci*, and *Pseudomonas* species. Commonly, initial treatment for infected necrosis includes broad-spectrum intravenous antibiotics with suitable penetration into the pancreas, like carbapenems, quinolones, metronidazole, and third- or higher-generation cephalosporins [17]; however, worsening infection may need earlier drainage and or debridement.

- Presence of symptoms, such as abdominal pain due to mass effect from large WOPN, nausea, vomiting, early satiety, and stomach discomfort due to gastric compression, intestinal, or biliary obstruction [18].
- Abdominal compartment syndrome. Intra-abdominal hypertension (IAH) and abdominal compartment syndrome (ACS) are not common and underrecognized complications of severe acute pancreatitis with high morbidity and mortality. The treatment of IAH/ACS requires a multi-modality approach; if conservative management is not effective, percutaneous or surgical decompression is warranted [19].

## 3. Timing of Drainage of PFCs

Current guidelines suggest delaying the first intervention for 4 weeks to allow for encapsulation and clearer delineation of WOPN margins, as well as to potentially prevent adverse effects if drainage is performed [7,20]. Nevertheless, the risks and benefits of earlier drainage before 4 weeks remain largely unexplored. Trikudanathan et al. [21], comparing interventions before and after 4 weeks of pancreatitis onset using an endoscopically centered step-up strategy, found early interventions (<4 weeks) were more frequently performed for infection and organ failure. While complications were similar, there was a slightly higher need for surgery with no significant difference in mortality. The study suggested considering early endoscopic drainage ± necrosectomy when there is a strong indication for drainage. Any early interventions for draining walled-off PFCs were examined in a meta-analysis, and the results revealed that early treatments (<4 weeks) were linked to higher death rates and did not reduce adverse events or improve clinical success [22]. However, a systematic review and meta-analysis by Ramai et al. [23] evaluating EUS-guided drainage of walled-off pancreatic fluid collections <4 weeks after development (182 patients, 28.9%) compared with ≥4 weeks after development (448 patients, 71.1%) indicated similar technical and clinical outcomes between the two groups (*p* = 0.11). There were no statistically significant differences in overall adverse events or mortality, while hospital stay was longer for patients undergoing early drainage compared with standard drainage. The study concluded that patients who need endoscopic drainage for severe acute pancreatitis complicated by walled-off pancreatic collections should not be delayed for 4 weeks.

## 4. The “Step-Up Approach” for the Management of WOPN

The current international guidelines propose the utilization of the “step-up approach”, advocating for a gradual escalation from a minimally invasive to a more invasive procedure. The “step-up approach” is indeed a staged management approach that involves endoscopic transluminal drainage (ETD) or percutaneous drainage (PCD) as first-line interventions. If required, additional and more aggressive interventions, including direct endoscopic necrosectomy (DEN) or minimally invasive surgical necrosectomy, are considered. A step-up strategy for infected PFCs was originally proposed in the PANTER trial, a randomized control trial, which was reported by the Dutch Pancreatitis Study Group [24]. Patients with infected PFCs were randomly assigned to have either an open necrosectomy or a step-up approach that consisted of minimally invasive methods such as percutaneous or endoscopic trans gastric drainage, switched to minimally invasive retroperitoneal necrosectomy if there was no clinical improvement. The step-up approach demonstrated significantly fewer major complications (*p* = 0.006) and decreased healthcare utilization (*p* = 0.004), while there was no significant difference in mortality. Afterward, in 2017, in a Dutch multicenter, randomized, superiority trial (TENSION trial), 98 patients with infected necrotizing pancreatitis and an indication for invasive intervention were enrolled and randomly assigned to the endoscopic step-up approach (endoscopic ultrasound-guided transluminal drainage followed, if necessary, by endoscopic necrosectomy, *n* = 51) or the surgical step-up approach (percutaneous catheter drainage followed, if necessary, by video-assisted retroperitoneal debridement, *n* = 47). At 6 months of follow-up, his section may be divided by subheadings. It should provide a concise and precise description of the experimental results, their interpretation, as well as the experimental conclusions that can be drawn. The endoscopic step-up approach was not superior to the surgical step-up approach in reducing major complications or death; the rate of pancreatic fistulas and length of hospital stay were lower in the endoscopy group [25]. These favorable short-term outcomes were confirmed by a single-center randomized clinical trial of 66 patients with confirmed or suspected infected necrotizing pancreatitis, randomly assigned to groups that received minimally invasive surgery (laparoscopic or video-assisted retroperitoneal debridement, *n* = 32) or an endoscopic step-up approach (transluminal drainage with or without necrosectomy, *n* = 34); the endoscopic approach significantly reduced major complications, lowered costs, and increased quality of life [26]. The TENSION trial evolved in the ExTENSION trial [27], published in 2022, where the authors evaluated the same endpoint of the TENSION trial in a longer period of follow-up of at least 5 years. At long-term follow-up, the endoscopic step-up approach was not superior to the surgical step-up approach in reducing death or major complications; however, patients assigned to the endoscopic group developed overall fewer pancreaticocutaneous fistulas and needed fewer reinterventions after the initial 6-months follow-up. Based on these results, the endoscopic approach with endosonography-guided transmural drainage and debridement, when feasible, is nowadays the procedure of choice in the minimally invasive management of pancreatic necrosis.

## 5. Evolution in Endoscopic Drainage Techniques

Endoscopic drainage of PFCs was first reported in 1989 with conventional endoscopic transmural drainage (EGD-TD). This technique consisted of endoscopic visualization of gastric or duodenal bulging secondary to PFC compression, with subsequent puncture of the gastrointestinal wall to create a cystogastrostomy tract, followed by tract dilation and plastic stent placement [28]. Even if this kind of drainage is safe and effective, only 40–50% of PFCs give a physical gastric or duodenal bulge to guide tract creation [29]. Endoscopic ultrasound-guided transmural drainage (EUS-TD) allows for direct visualization of the PFC, identification of blood vessels or necrosis, and aspiration of PFC fluid to confirm placement within the collection, thus allowing for a higher rate of technical success in drainage [30]. Indeed, a prospective randomized trial reported a clinical success rate of 100% for EUS-TD versus 33% for CTD [31]; therefore, current guidelines recommend EUS-TD as the endoscopic technique of choice for PFC drainage [7,8].

**Figure 2:**
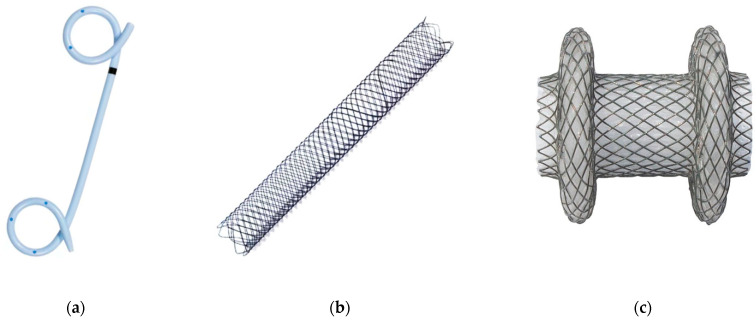
(**a**) Double pigtail plastic stent (DPPS); (**b**) fully covered self-expandable metal stent (FCSEMS); (**c**) lumen-apposing metal stent (LAMS).

Traditionally, double pigtail plastic stents (DPPSs) were the mainstay of therapy for EUS-TD (Figure 2a). DPPSs are cheap, safe, widely accessible, and easily removable, but they require multiple stent placement and dilation procedures to obtain a cystoenterostomy of caliber wide enough to perform DEN. Thus, despite high efficacy for drainage of pseudocysts (85.1–90.8%) [32], WOPN resolution with DPPS drainage alone shows a clinical success between 30.8% and 52.1% [25,33,34].

Fully covered self-expandable metal stents (FCSEMSs) became increasingly popular thanks to their advantage of having a wider lumen than DPPSs, which allows better drainage of solid debris with lower rates of occlusion (Figure 2b) [35,36]. A prospective case series published by Talreja et al. [37] reported a 95% clinical success rate for FCSEMSs in draining PFCs. Nevertheless, their tubular design and absence of anti-migration properties led to a high risk of migration, causing potential injuries to the duodenal/gastric wall or retroperitoneum, resulting in bleeding and/or perforation, which hindered their widespread use [38].

To overcome the limits of DPPSs and FCSEMSs, in 2011, a different SEMS was developed, called lumen-apposing metal stent (LAMS) and characterized by a “dumbbell” shape given by two bilateral double-walled anchoring flanges with a 90◦ angle between each flange and the narrower central part of the stent, made of braided nitinol, fully covered with silicone (Figure 2c) [39]. The AXIOS stent (Boston Scientific, Marlborough, MA, USA) was the first LAMS commercialized; subsequently, other LAMSs and SEMSs, equipped with anti-migratory systems and varying in shapes and features, were introduced by different manufacturers (Spaxus and Nagi, by Taewoong Medical, Gimposi, Korea; Z-EUS IT™, by M.I.Tech, Pyongtaek, Korea).

LAMSs have revolutionized the endoscopic management of WOPN due to the advantages led by their design: the shorter length and biflanged shape prevent migration. The wider diameter allows for more effective drainage of solid necrotic debris, preventing stent occlusion, and makes it easier to enter the PFC cavity for direct endoscopic necrosectomy (DEN) [40].

**Figure 3:**
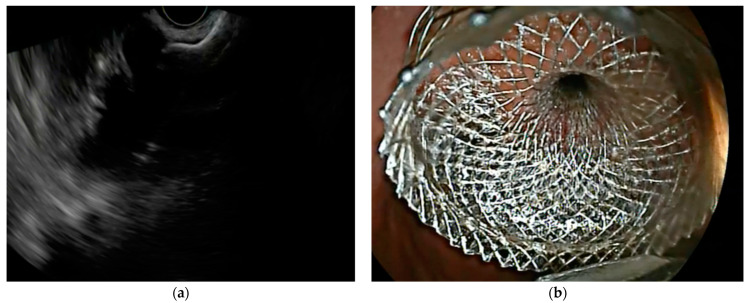
EUS (red arrow) (**a**) and endoscopic (**b**) view of a large-caliber LAMS immediately after its deployment in order to drain a WOPN.

Furthermore, the electrocautery-enhanced LAMS (HOT-AXIOS, Boston Scientific, Marlborough, MA, USA), developed in 2015, has the added benefit of an electrocautery-enhanced delivery system that allows the catheter and LAMS to be passed into the PFC with a single EUS-guided puncture, eliminating the need for tract dilatation, guidewire exchanges, or fluoroscopy. Bekkali et al. [41] confirmed that the use of EC-LAMSs was associated with a reduced drainage time when compared with “cold”-LAMSs; thus, they are currently the most widely used in clinical practice. Several studies have demonstrated high technical success rates (95–100%) and clinical success rates (84.2–86.3%) in EUS-guided drainage of WOPN using LAMSs [40,42]. The EC-LAMS is available in many different diameters and lengths, extending up to 20 mm [43]. Logically, a larger LAMS diameter promotes better output of solid necrotic debris and facilitates easier access for DEN with larger therapeutic endoscopes. An international multicenter retrospective study conducted by Parsa et al. [44], comparing the 15 and 20 mm EC-LAMSs, confirmed that fewer DEN sessions are needed to reach WOPN resolution when using the 20 mm EC-LAMS (mean 1.3 vs. 2.1; *p* < 0.001), with similar clinical success rates (92.2% vs. 91.7%, odds ratio 0.92; *p* = 0.91), overall adverse events (21.6% vs. 15.2%; *p* = 0.72), and bleeding events (4.9% vs. 3.4%; *p* = 0.54). Consequently, 20 mm LAMS placement could potentially offer advantages in managing more complex and larger WOPN cases (Figure 3).

## 6. Type of Stents

International guidelines are not consistent regarding the use of LAMSs. While the ESGE guidelines suggest that both LAMSs and BPPSs can be considered [7], the AGA guidelines conclude that LAMSs are preferred [8]. A survey found that 16 of 22 advanced endoscopists believed that LAMSs should be the standard of care for WOPN [45]. Moreover, at present, strong recommendations cannot be made regarding the use of one type of stent over another, and current data published in the literature are conflicting, especially when comparing LAMSs and DPPSs.

Siddiqui et al. [46] reported the superiority of LAMSs over FCSEMSs in terms of a lesser number of procedures for WON resolution (2.2 vs. 3), reduced need for endoscopic reintervention following stent occlusion (3.5% vs. 21.5%), and lower stent migration rate (0% vs. 5.8%). There was no significant difference in technical and clinical success rates; however, early adverse events were more frequent in the LAMS group (OR, 6.6).

A retrospective study comparing 30 patients drained with EC-LAMSs and 60 patients with biliary FCSEMSs reported a higher clinical success with a tendency to significance in patients drained with an EC-LAMS (96% vs. 82%, *p* = 0.055) and a lower adverse event rate (4% vs. 18%, *p* = 0.04). Moreover, stent deployment time was significantly reduced in the EC-LAMS group [47]**.**

An RCT by Bang et al. [48] that compared the efficacy of LAMSs and plastic stents for WOPN drainage showed no difference in the total number of procedures, length of hospital stay, or overall treatment costs. Although the procedure duration was shorter, procedure costs and stent-related adverse events were higher with LAMSs; indeed, the trial results raised important safety concerns, as LAMSs were associated with higher stent-related complications if not removed within 3 weeks.

Boxhoorn et al. [49] found that the need for endoscopic transluminal necrosectomy in patients with infected necrotizing pancreatitis treated with a LAMS was not lower compared with plastic stents. Clinical outcomes, including the total number of interventions, length of hospital stay, and total healthcare costs, as well as complications (especially bleeding), did not differ either between groups. The authors concluded that LAMSs and plastic stents can both be used for endoscopic transluminal drainage of infected necrosis.

On the other hand, a large retrospective study by Chen et al. [50], including 189 WOPN, reported that drainage using LAMSs is associated with higher clinical success, shorter procedure time, lower need for surgery, and lower rate of recurrence when compared to DPPSs. A cost-effectiveness analysis comparing LAMSs with DPPSs in the management of WOPN reported that, despite the higher cost of LAMSs, their higher efficacy makes this device more cost-effective [51].

A systematic review and meta-analysis [52] of data from three randomized trials, including 206 patients, reported that, except for procedure duration, which was significantly shorter for LAMSs, there was no significant difference in the need for necrosectomy, number of interventions, treatment success, recurrence, readmission, length of hospitalization, mortality, bleeding procedural adverse events, or overall costs between LAMSs and DPPSs, respectively.

Given that clinical and technical outcomes appear to be similar among LAMSs and DPPSs, the decision to use either method is typically driven by endoscopists’ experience and availability. However, it is important to stress that not all patients affected by PFCs need LAMSs, but they can be treated with multiple DPPSs. Conversely, LAMSs should be the stent of choice in case of large-sized WON with an amount of necrotic debris requiring subsequent DEN [53].

## 7. Coaxial Placement of a Double Pigtail Plastic Stent through an LAMS

Coaxial placement of a double pigtail plastic stent through an LAMS has been proposed as a method to reduce the typical LAMS adverse events such as bleeding or stent occlusion, but its real utility is still under debate and not clearly demonstrated. Indeed, it has been hypothesized that the placement of the coaxial DPPS through the LAMS could have a protective effect in preventing the friction of the distal flange against the adjacent wall of the cavity, which would reduce the risk of bleeding [54]. A study by Puga et al. [55] demonstrated that adjunctive placement of DPPSs resulted in decreased adverse events, particularly bleeding, while AbiMansour et al. [56] found there were no significant differences in rates of clinical success (75.9% LAMS vs. 69.6% LAMS/DDPS, *p* = 0.34) or overall AEs (15.7% LAMS vs. 15.7% LAMS/DPPS, *p* = 0.825). Another potential benefit of coaxial DPPSs is the frequency of food impaction inside the collection cavity, minimizing the risk of infection: this hypothesis finds support in the findings of Aburajab et al.’s study [57], who reported a trend toward higher infection with LAMSs alone.

A meta-analysis by Giri et al. [58] analyzed eight studies from 2010 and 2022 and showed that, although the overall AE rate was lower in the LAMS-DPPS group, the results were not statistically significant, and the effort and cost that is involved in the placement of an additional coaxial stent do not translate to better clinical outcomes. Furthermore, these results conflict with those of the first randomized controlled trial by Vanek et al. [59], including 67 patients randomly assigned to LAMS placement with (*n* = 34) or without (*n* = 33) DPPS. The addition of a coaxial DPPS was associated with a lower global adverse event rate (20.7% vs. 51.5%, respectively; *p* = 0.008) and stent occlusion (14.7% vs. 36.3%, *p* = 0.042). Larger RCTs are required to demonstrate the benefit of routinary coaxial DPPS placement within LAMSs in clinical practice.

## 8. Multiple Transluminal Gateway Technique (MTGT)

**Figure 4:**
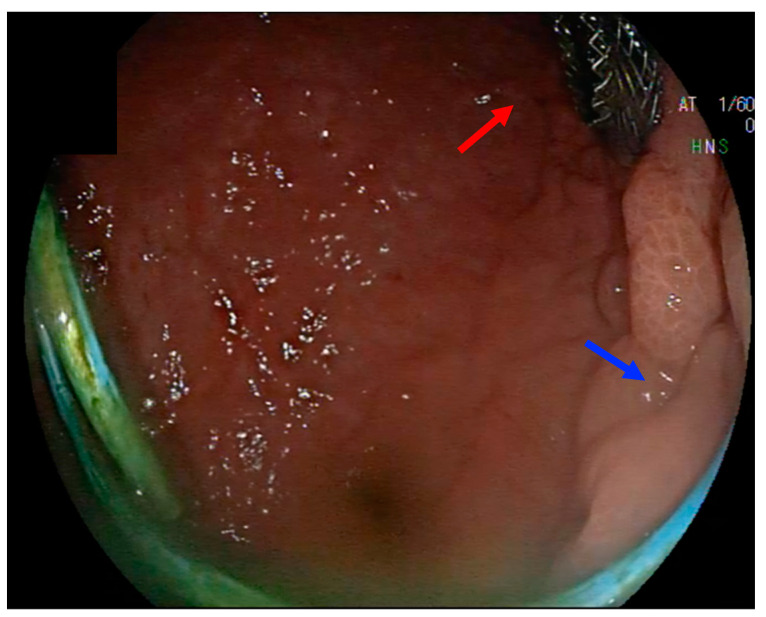
Modified multiple transluminal gateway technique (MTGT): large WOPN drained with an LAMS deployed at the level of the distal gastric body (red arrow) and a DPPS placed at the proximal body/cardia (blue arrow).

The multiple transluminal gateway technique (MTGT) consists of the placement of multiple stents between the necrotic cavity and gastrointestinal lumen for better drainage of necrotic collections. This technique was first described by Varadarajulu et al. [33] in 2011: multiple DPPSs were placed under EUS guidance in order to create 2–3 transmural fistulas. The authors reported treatment success in 11/12 (91.7%) patients treated with MTGT (in comparison to 25/48 (52.1%) patients who underwent the single gateway technique (SGT)). The need for endoscopic necrosectomy and surgery was lesser in the MTGT group. Commonly, MTGT is used when the cavity is complex, bigger than 12 cm, or multiloculated. In a case series by Binda et al. [60], the authors evaluated the feasibility and safety of the single-step MTGT technique using LAMSs for six patients with complex WOPN. The technical success rate was 100%, and there were no cases of residual necrosis or WOPN recurrence; despite the small number of patients, the authors concluded that for complex WOPN, the single-step MTGT with electrocautery-LAMSs could be a feasible and well-tolerated treatment option. A modified MTGT has been proposed by the group of Varadarajulu in their Orlando Protocol for endoscopic management of PFCs in the era of LAMSs [61]: in case of WON > 10 cm and disconnected pancreatic duct syndrome (DPDS), the technique comprised the placement of LAMSs at the level of the body/antrum to facilitate access for DEN, associating two other DPPSs placed proximally (proximal body or cardia), corresponding to the body/tail of the pancreas (Figure 4). After 3–4 weeks, the LAMS was removed, and the DPPSs were left indwelled indefinitely to avoid PFC recurrence.

ESGE guidelines suggest that MTGT should be taken into consideration in patients who have multiple or large (>12 cm) walled-off necrosis or in cases where single transluminal gateway drainage does not perform as well as desired [7]. Moreover, additional access is sometimes necessary when the first access is in such a position that it does not allow easy scope introduction into the cavity for DEN.

## 9. Dual-Modality Drainage

Dual-modality drainage (DMD) is represented by a combination of endoscopic transluminal drainage (ETD) or percutaneous drainage (PCD), developed for the management of larger and more complex WOPNs. This approach was first introduced by Ross et al. [62] in 2014 with the aim of reducing the high rate of chronic pancreatocutaneous fistula and the need for surgery in patients with disconnected pancreatic duct syndrome (DPDS). Ross et al. [62] treated 117 patients with symptomatic and infected WOPN. Transluminal stents were placed endoscopically into the collection immediately after percutaneous drainage; this allowed redirection of pancreatic juice into the GI tract rather than through the skin, thus decreasing the risk of chronic pancreatocutaneous fistula formation. The rate of pancreaticocutaneous fistulae was 0%, with no need for surgical necrosectomy or surgical treatment and no procedure-related deaths. Even if EUS-guided transmural (transgastric/transduodenal) drainage with lumen-apposing metal stents or plastic stents along with endoscopic necrosectomy is the preferred modality for the drainage of WOPN, in some situations, it is not technically feasible, therefore adding percutaneous drainage is of crucial importance in these patients. Moreover, percutaneous catheter placement into the retroperitoneum and/or pelvis facilitates drainage of these endoscopic-undrainable areas and allows for bedside irrigation and clearance of necrotic material. ESGE guidelines [7] suggest considering concurrent endoscopic transmural drainage and percutaneous drainage in patients with walled-off necrosis with extension to the pelvic paracolic gutters.

## 10. Direct Endoscopic Necrosectomy

Following EUS-transmural drainage, a conventional forward-viewing endoscope can be inserted through the cystogastric/cystoenteric fistula, and the WOPN cavity can be accessed “directly” in order to perform debridement of necrotic material from the cavity. Endoscopic necrosectomy consists of a combination of sucking debris through the working channel, removing necrotic material with a removal device, and applying irrigation. Because of its lower complication rates, shorter hospital stays, and excellent clinical success rates, DEN is the preferred treatment modality for WOPN [40]. Multiple DEN sessions are usually necessary to obtain the complete resolution of WON; in a meta-analysis, the mean number of DEN sessions varied from 1 to 15 with a weighted mean of 4.09 procedures [63]. A novel classification system for WON, known as the QNI classification system, was recently proposed by Baroud et al. [64]. It is based on the number of abdominal quadrants (“Q”) afflicted by WON, the percentage of necrosis of WON (referred to as “N”), and the presence of infection (“I”). Patients were divided into two groups: those with a lower QNI stratification (2 quadrants and 30% necrosis; group 1) and those with a higher stratification (≥3 quadrants, 2 quadrants with ≥30% necrosis, or 1 quadrant with >60% necrosis and infection; group 2). Individuals in group 2 required more DEN sessions (2 vs. 1, *p* = 0.001) and had longer hospital admissions, a longer mean time to resolution (79.6 vs. 48.4 days, *p* = 0.02), and a higher mortality rate.

DEN could be performed as a step-up approach only in patients who have not responded to drainage alone or in the same session immediately after LAMS placement. In a multicentric retrospective study, Yan L et al. compared the clinical outcomes and predictors of success for endoscopic drainage of WON with LAMSs followed by immediate or delayed DEN, concluding that performing DEN at the time of stent placement was an independent predictor for resolution of WON with lesser number of DEN sessions (3.1 vs. 3.9, *p* < 0.001), with comparable AEs [65]. These findings have been supported by the results of the recent DESTIN RCT [66]: in patients with confirmed or suspected infected necrotizing pancreatitis, an approach incorporating upfront necrosectomy at the index intervention rather than as a step-up measure could safely reduce the number of reinterventions required to achieve treatment success, without statistically significant difference in overall disease-related adverse events or mortality.

**Figure 5:**
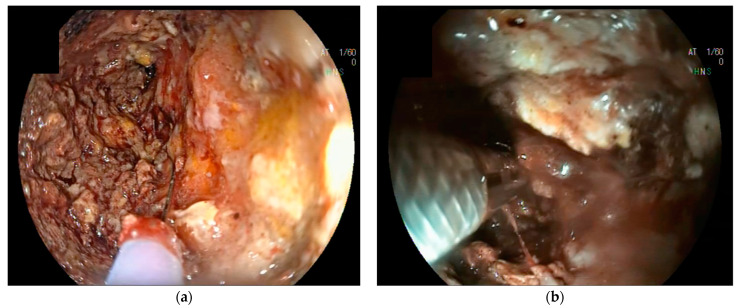
Direct endoscopic necrosectomy (DEN) using a conventional snare (**a**) and using a dedicated device, the new EndoRotor Powered Endoscopic Debridement (PED) System^®^ (**b**).

The major limitation of this procedure is the lack of dedicated devices, and commonly, a variety of auxiliary instruments designed for other indications like snares, baskets, nets, stone extraction balloons, and forceps have been used for DEN. ESGE guidelines [7] suggest that snares and baskets might be preferred for the primary attempt as they are safe and quite effective (Figure 5a). Due to different consistencies of necrosis inside the cavity, the use of different devices during the same DEN session could often be necessary. Necrotic debris has to be grasped and released outside the cavity, requiring g repeated passages of the endoscope through the cystogastric fistula. For all these reasons, DEN is a time-consuming procedure, and effort has been recently made to develop novel dedicated devices to simplify and speed up this procedure. EndoRotor Powered Endoscopic Debridement (PED) System^®^ (Interscope Medical, Inc., Worcester, MA, USA) is a new through-the-scope device that can simultaneously suck and cut the necrotic debris using negative pressure allowing to remove the necrosis without the necessity to go in and out the cavity and to change many devices (Figure 5b). The Over-The-Scope-Grasper (OTSG XcavatorTM–Ovesco Endoscopy AG, Tübingen, Germany) is a grasper mounted on the tip of the endoscope that can remove up to 1 cm3 of necrotic tissue and discharge it into the stomach, leaving the working channel free for irrigation and aspiration. Necrolit^®^ (Meditalia SAS, Palermo, Italy) is a new time-saver device composed of an atraumatic ultrastiff monofilament snare and a no-tip Dormia basket, running together in a single catheter. These new devices have shown promising results in small case series, but prospective trials are ongoing to evaluate their safety and efficacy [67,68,69].

## 11. Role of Discontinuation of Proton Pump Inhibitors

There are no specific guidelines about the medical management of patients undergoing direct necrosectomy for the treatment of WON; in particular, the use of proton pump inhibitors is still unclear, and the available literature on this topic is poor. Some clinicians use PPI because gastric acid suppression may lower the rates of certain adverse events, including bleeding and gastric ulceration. However, other authors suggest that suppression of the acidic gastric contents with PPI could reduce the rate of dissolution of solid necrotic debris. A multicentric retrospective study by Powers et al. [70] was the first study to investigate the utility of PPI therapy in patients undergoing direct necrosectomy. The PPI and non-PPI groups had similar clinical success rates, but a significant difference in the required number of direct endoscopic necrosectomies has been highlighted between the two groups (PPI groups 4.6 vs. non-PPI group 3.2, *p* < 0.01). Moreover, the two groups had similar rates of adverse events, but there were significantly more cases of stent occlusion in the non-PPI group vs. the PPI group (20.1% vs. 9.5%, *p* = 0.012). The evidence to support PPI therapy discontinuation is limited, and further trials are needed to confirm these results; therefore, in clinical practice, PPI should be stopped only when there is no strong indication to continue its use.

## 12. Chemical Debridement

Hydrogen peroxide (H_2_O_2_) is a colorless liquid that, when it comes into contact with organic tissue, quickly breaks down into water and oxygen, creating a soft foam that aids in the removal of materials attached to the tissue, such as necrotic debris [71,72]. Moreover, it has antiseptic and sporicidal properties and causes irritation of the wall of the PFC, leading to the formation of granulation tissue and fibrosis, resulting in obliteration of the PFC cavity [73]. Usually, a 3% H_2_O_2_ solution is diluted in a 1:1, 2:1, or 3:1 saline solution, and a variable quantity from 100 mL to 300 mL is delivered and directed into the WOPN at the end of DEN.

Recently, multiple studies have reported the use of H_2_O_2_ irrigation for the treatment of WOPN, reporting decreased procedure time and shorter time to resolution. A large multicenter retrospective study comparing irrigation with H_2_O_2_ (*n* = 122) and standard irrigation without H_2_O_2_ (*n* = 82) during DEN for WOPN demonstrated that H_2_O_2_-assisted endoscopic necrosectomy had a higher clinical success rate (93.8% vs. 78.9%, *p* = 0.002), with no difference in adverse events. On a multivariate analysis, the use of H_2_O_2_ was associated with higher clinical success rate (odds ratio 3.30, *p* = 0.033) and earlier resolution (odds ratio 2.27, *p* < 0.001) [74].

A meta-analysis by Garg et al. [75] reported a high technical and clinical success rate (97.3% and 89.8%, respectively), with an AE rate of 17.9% comparable to DEN performed without H_2_O_2_.

A severe complication correlated with H_2_O_2_ use in neurosurgery, orthopedic surgery, fistula irrigation, and wound debridement is gas embolism [76,77,78,79]. A potentially lethal AE may be due to the rapid and high amount of free oxygen released after contact with organic tissue. To the best of our knowledge, no cases of such adverse events have been reported in the published series of H_2_O_2_-assisted DEN.

Future well-designed randomized controlled studies are needed in order to establish the role of H_2_O_2_ in pancreatic WOPN debridement and investigate the optimal technique, concentration, and best predictors of success.

## 13. LAMS-Related Adverse Events in PFC Management

EUS-guided PFC drainage is considered a safe and effective procedure, although AEs are not rare, and their management is not standardized. The overall LAMS-related AE rate treating WOPN appears difficult to evaluate because of the lack of data uniformity and different definitions among published series, but it is generally between 10 and 25% [80,81].

In a multicenter, international, retrospective analysis of adverse events associated with LAMS placement for PFCs, Fugazza et al. [82] reported an overall adverse events rate of 24.3% (74/304 patients). The most frequent LAMS-related AE was of bleeding (27.8%), followed by stent migration (25.3%), infection (24.1%), stent occlusion (17.7%), buried stent syndrome (3.8%) and occlusion of the pylorus (1.3%). According to the ASGE lexicon [83], most of the AEs were classified as mild or moderate and 6.3% as severe. A total of 58.2% were managed endoscopically, 34.2% were managed conservatively, and 7.6% were managed through interventional radiology. No AE required surgical management.

AEs are well known and described in many studies, although standardized endoscopic measures of management have not been addressed and are generally based on expert opinions.

Bleeding is one of the most common adverse events, with an overall risk of 7.2% (22/304) and severe bleeding occurring in 0.98% of cases [82].

PFC-related bleeding could occur during EUS-drainage or subsequent DEN sessions and can originate at the puncture site from small missed venous collaterals of gastric or duodenal wall, from minor vessels within the cavity, or from large retroperitoneal vessels that may bleed after the rapid collapse of the collection or due to iatrogenic damage during necrosectomy [84]. Instead, delayed bleeding is usually caused by prolonged contact between the distal flange of the indwelling LAMS and the walls, which can lead to the erosion of intracavitary vessels and promote the formation of a pseudoaneurysm [84]. Non-severe bleeding is generally self-limited and usually originates from the gastric wall and can eventually be managed with classical hemostatic techniques. When bleeding originates inside the cavity, usually from the rupture of a pseudo aneurism of a large retroperitoneal vessel, there are low possibilities of successful endoscopic hemostasis, often requiring radiologic embolization.

To prevent the risk of delayed bleeding related to LAMS erosion of the cystic wall, the LAMS has to be removed about 4 weeks after deployment, but a residual bleeding rate of 3.5% is still reported [85].

The use of a co-axial DPPS within the LAMS has been proposed as another strategy to prevent the risk of bleeding, even though its real efficacy is still under debate [55].

Binda et al. [86] reported a case series of 10 patients treated with PuraStat ^®^ (3-D Matrix Europe SAS, Caluire-et-Cuire, France), a novel hemostatic peptide gel, both prevention and management of PFC-related bleeding. Indeed, PuraStat can be easily and safely applied inside the cavity of the WOPN, is effective in treating oozing-type bleeding, and, thanks to its three-dimensional structure, might promote healing, preventing delayed bleeding.

A recent systematic review [87] reported a 2.0% frequency of LAMS misdeployment during EUS-guided PFC drainage. Distal flange misdeployment in the peritoneal cavity, external to the cystic wall, was reported in up to 36.1% of cases, managed in most cases with over-the-wire placement of a new stent (LAMS, SEMS, or double pigtail stents/plastic) through the novel fistula tract as a rescue therapy. Two cases of complete LAMS misdeployment inside the PFC were reported: the misdeployed LAMS was left inside the PFC, and a new LAMS was deployed through the fistula. Both stents were successfully removed four weeks later [88].

LAMSs were originally designed with a biflanged shape to minimize the risk of stent migration, but LAMS dislodgements, in particular during DEN, are reported. One study has shown that performing a direct endoscopic necrosectomy at the time of LAMS insertion is associated with a greater chance of stent dislodgement compared to performing endoscopic necrosectomy after 1 week (4.3% vs. 0%) [65].

Buried LAMS syndrome is a rare LAMS-related AE that occurs when a gastric or enteric tissue overgrowth due to persistent inflammation buries the proximal flange, which becomes no longer identifiable endoscopically [89]. A systematic review of AEs after PFC drainage has reported buried stents in 4 out of 14 studies [90], and a large retrospective study reported a buried LAMS occurrence rate of less than 10% [91]. It usually develops due to longer left in situ of the LAMS, often more than 6 weeks, and this underscores again the importance of removing the LAMS after 4 weeks in order to reduce the adverse events. Also, the location in the gastric cavity can influence the development of a buried stent syndrome; in fact, it has been proposed that the increased motility of the gastric antrum results in more vigorous traction on the stent, producing a hypertrophic inflammatory response and a higher risk of buried LAMS syndrome [92]. Treating this rare complication is challenging, and specific indications on the technique of choice exist and can be associated with adverse events like massive bleeding. If the fistulous tract is still visible, it can be cannulated with a catheter and guidewire and eventually dilated using a 10 mm balloon; then, the distal flange can be grasped with force and removed by withdrawal and inversion [89]. One potential option when a fistulous tract is evident is to insert a second LAMS into the occluded and buried LAMS [93]. When the fistulous tract is no longer visible and cannulation is impossible, a needle knife sphincterotome or argon plasma coagulation can be used to unroof the gastric mucosa [94].

LAMS occlusion due to food impaction or solid debris is another well-known complication that leads to a risk of overinfection of the undrained collection, a longer time for resolution, and a lower rate of clinical success. Most of the time, it can be treated endoscopically by declogging the LAMS using a snare, basket, or stone-extraction balloon. As shown in the study of Vanek et al. [59], the use of a co-axial DPPS might be a valid strategy to reduce the rate of LAMS occlusion (14.7% in the LAMS+ co-axial DPPS group vs. 36.3% in the LAMS alone group, *p* = 0.042).

## 14. Conclusions

Symptomatic and infected necrotic collections still represent life-threatening complications of severe acute pancreatitis, often requiring a complex and multidisciplinary approach. The EUS-guided transluminal step-up approach has been proven to be a safe and effective method to manage these complications, reducing the need for open surgical necrosectomy. Even though the introduction of LAMSs has facilitated the treatment of WON, they do not reduce the number of necrosectomy sessions and have an important rate of complications, demanding an expert approach. To conclude, the best strategy to manage WON is still evolving, and further research is needed to determine the best approach for each patient.

## Data Availability

All articles cited in this article are listed in PubMed.
